# Laparoscopic Right Colectomy for Cecal Diverticulitis With Inguinal Abscess: A Rare Case Report

**DOI:** 10.1111/ases.70140

**Published:** 2025-08-25

**Authors:** Koki Miya, Fumitaka Yagi, Takeshi Kato, Takashi Saitoh, Katsuhiko Suzuki

**Affiliations:** ^1^ Department of General Surgery Honjo Daiichi Hospital Akita Japan

**Keywords:** cecal diverticulitis, inguinal abscess, laparoscopic right colectomy

## Abstract

Right‐sided colonic diverticulitis is generally considered less prone to severe complications than left‐sided colonic diverticulitis; progression to an inguinal subcutaneous abscess via retroperitoneal extension is extremely rare. Herein, we report a unique case of cecal diverticulitis, wherein a retroperitoneal abscess extended into the inguinal region. A 65‐year‐old man presented with right inguinal swelling. Imaging revealed an abscess of cecal diverticulitis with retroperitoneal tracking into the inguinal subcutaneous tissue. After initial treatment with antibiotics and drainage, an interval laparoscopic right colectomy was performed. Intraoperative findings comprised severe adhesions and a suspected fistula, which was confirmed by indigo carmine staining of the resected specimen. This case highlights not only the rarity of right‐sided colonic diverticulitis with inguinal extension, but also the clinical value of a reproducible, staged surgical strategy. Such a strategy—initial infection control followed by minimally invasive resection—demonstrates safety and effectiveness, even in anatomically challenging and atypical presentations.

## 1 Introduction

The retroperitoneum is the anatomical space located behind the peritoneum and includes several compartments, such as the anterior, perirenal, and posterior pararenal spaces; all of which are continuous with the pelvic floor and surrounded by the pelvic musculature.

When an abscess forms in the retroperitoneum, it may traverse along the pelvic musculature through the internal inguinal ring and extend into the inguinal region [1].

Colonic diverticulitis is an inflammatory condition resulting from infection or perforation of diverticula in the colon. Most cases in the literature report abscess extension to the inguinal subcutaneous tissue originating from the sigmoid colon, whereas inguinal subcutaneous abscesses caused by right‐sided colonic diverticulitis are extremely rare worldwide [2, 3].

In Asia, the incidence of colonic diverticulitis is increasing with aging populations, and it is known to predominantly affect the right colon [4]. Many cases improve with conservative treatment; however, when perforation or extensive abscess formation is present, surgical intervention may be required. Therefore, flexible management based on individual clinical scenarios is essential [5].

Herein, we report a case of an abscess caused by cecal diverticulitis that extended through the retroperitoneum to the inguinal subcutaneous tissue. Conservative management was initially chosen, followed by laparoscopic right colectomy after inflammation resolution. This case demonstrates the rarity of right‐sided diverticulitis with inguinal extension and the value of a staged surgical strategy. This approach, involving initial infection control followed by minimally invasive resection, has proven to be safe and effective, even in challenging manifestations. The case provides a practical reference to managing similar future cases.

## 2 Case Presentation

**FIGURE 1:**
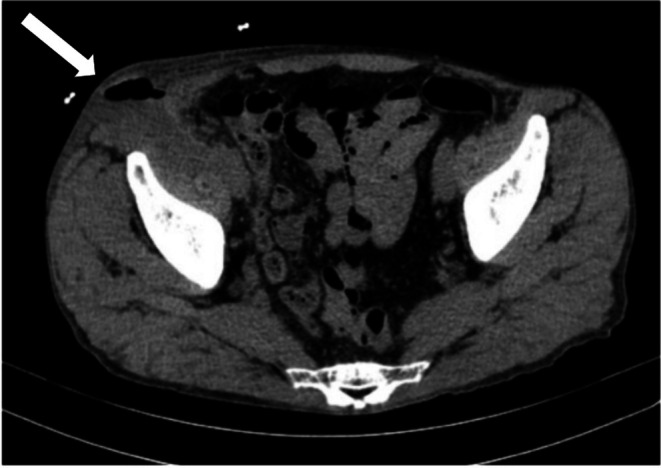
Plain abdominal CT scan. A low‐density area (white arrow) extending from the retroperitoneum near the cecum to the right inguinal region can be observed, suggesting abscess formation.

**FIGURE 2:**
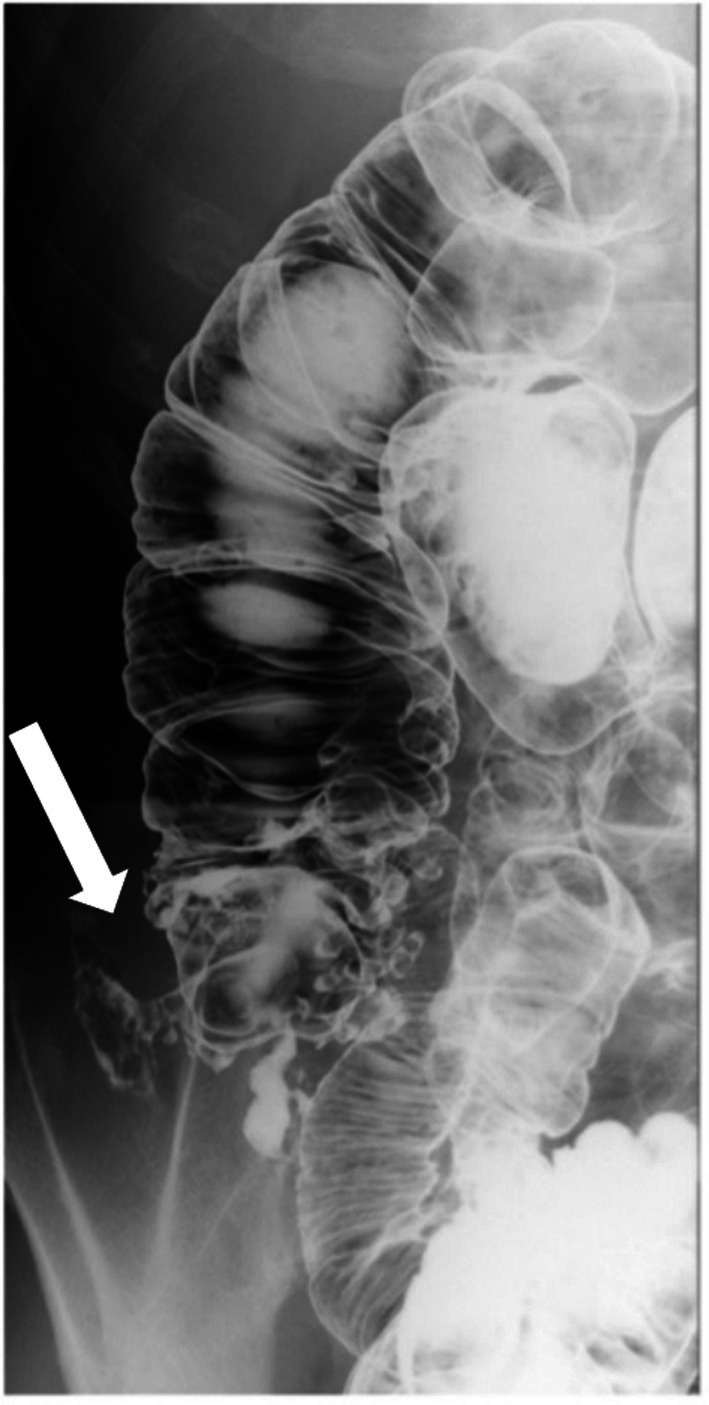
Contrast enema. Multiple diverticula were noted in the cecum with localized extraluminal contrast leakage (white arrow), suggestive of a fistula.

A 65‐year‐old man presented with swelling in the right inguinal region. Abdominal computed tomography (CT) revealed multiple diverticula from the cecum to the ascending colon and a low‐density area extending from the right retroperitoneum to the right inguinal subcutaneous area, suggesting abscess formation (Figure 1). Initial treatment included intravenous cefmetazole (1 g every 12 h for 10 days) and incision and drainage, which led to prompt defervescence and temporary clinical improvement, despite receiving no anti‐inflammatory medications. Cultures of the drained fluid revealed *Escherichia coli* , which was sensitive to cefmetazole. The antibiotic therapy was effective; no change in the antimicrobial agent was required. However, the patient repeatedly developed similar abscesses with each recurrence. Based on this clinical course, a fistulous connection between the cecal diverticulum and retroperitoneum was strongly suspected. Contrast enema also revealed findings suggestive of a fistula. Moreover, multiple diverticula were observed in the ascending colon (Figure 2). Therefore, laparoscopic right colectomy for the fistulous cecal diverticulum and surrounding diverticula was selected as definitive treatment to prevent further recurrence.

**FIGURE 3:**
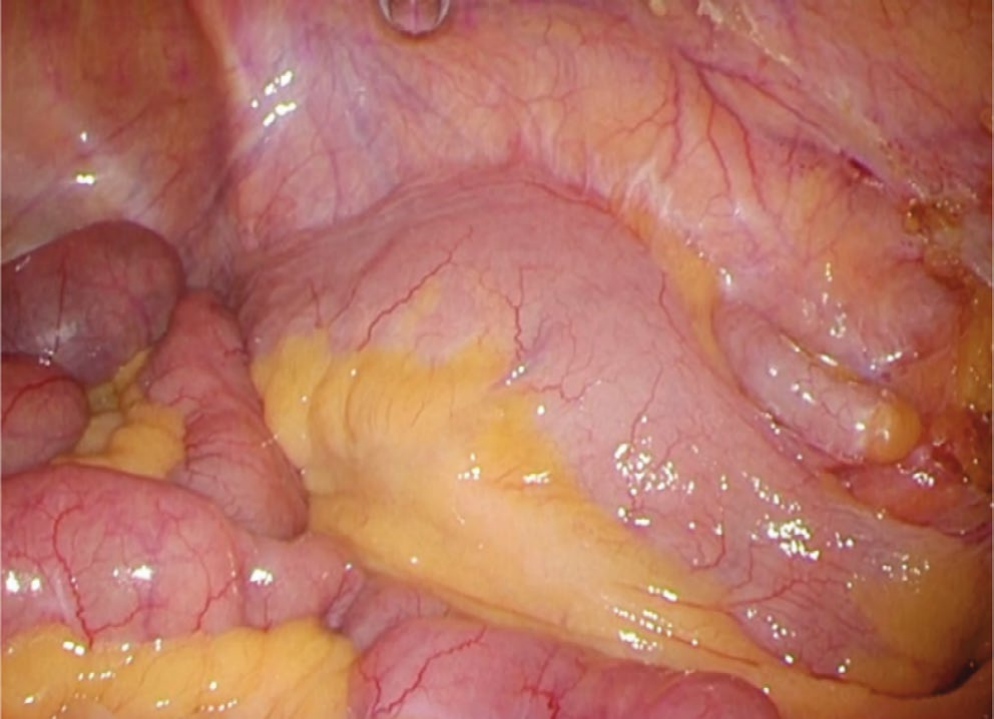
Operative laparoscopic view. Adhesions were observed around the cecum and abdominal wall. As inflammation had subsided with conservative therapy, tissue edema and fragility were mild, and the surgical field was relatively well maintained.

A five‐port technique was used with trocars placed at the umbilicus (12 mm), right and left lateral abdomen (5 mm each), right lower abdomen (12 mm), and left lower abdomen (5 mm). Indigo carmine dye was injected through the previous inguinal incision site. Adhesions were observed around the cecum, abdominal wall, and omentum. Since inflammation had subsided with conservative therapy, tissue edema and fragility were mild, and the surgical field was relatively well maintained (Figure 3). Strong adhesions were found around the suspected fistula site. Since important retroperitoneal structures such as the ureter and testicular vessels were present and should be preserved, particular attention was paid to avoid injury. Dense adhesions were noted on the lateral aspect of the cecum and ascending colon; therefore, a medial approach was employed to allow direct visualization and identification of these critical structures. Careful manipulation allowed safe mobilization from the retroperitoneum to the hepatic flexure without bowel injury. The right colon was exteriorized via a small laparotomy incision and resected from the terminal ileum to the ascending colon, and a functional end‐to‐end anastomosis was performed. The procedure was completed without intraoperative complications or the need for conversion to open surgery.

**FIGURE 4:**
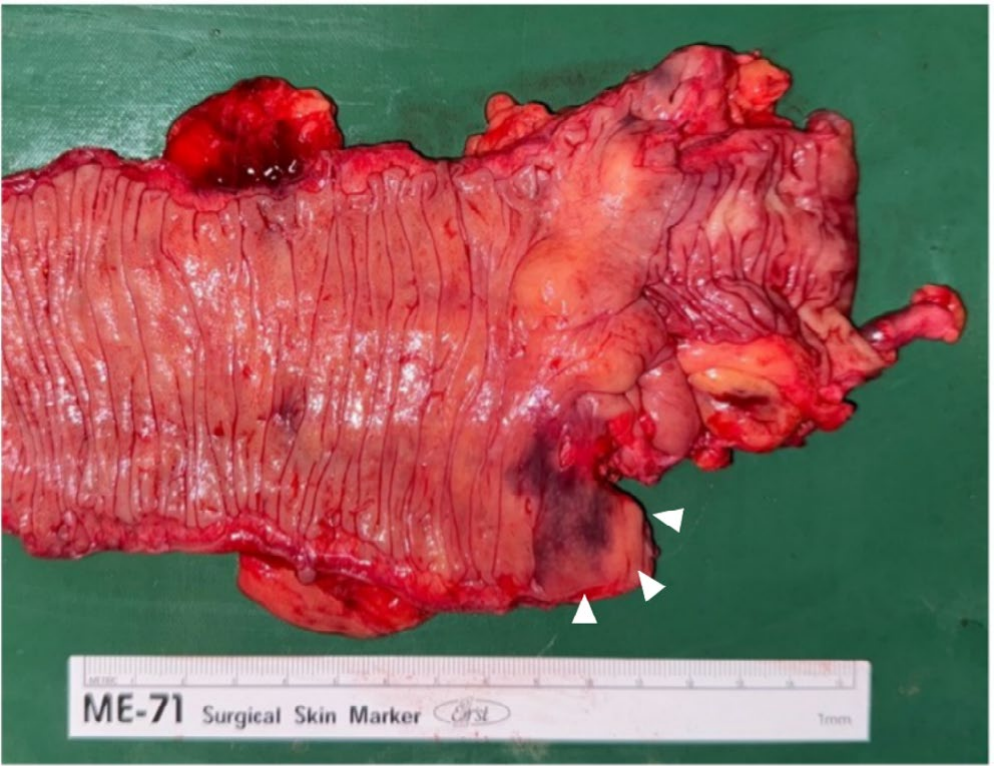
Gross findings of the resected specimen. Blue staining with indigo carmine was observed in the cecal diverticulum (white arrowhead), indicating the possible presence of a narrow fistula.

The resected specimen stained blue with indigo carmine at the cecal diverticulum, suggesting the presence of a tiny fistula (Figure 4). No immediate complications were observed, and the patient was discharged on postoperative day 11. However, 5 months after surgery, the patient presented with swelling in the right inguinal region, wherein a recurrent abscess was identified on CT. The contrast study via the inguinal route revealed no obvious communication with the intestinal tract. Therefore, the lesion was considered a residual abscess rather than recurrent diverticulitis. Preoperatively, the patient had developed an extensive fistula and abscess extending from the cecum through the retroperitoneal space to the subcutaneous inguinal region. Therefore, this residual fistulous tract was thought to have caused chronic inflammation, which manifested as a delayed postoperative complication. Since the primary lesion in the colon had already been resected, the condition was conservatively managed with intravenous cefmetazole (1 g every 12 h for 14 days) and percutaneous drainage. *Escherichia coli* was isolated from the drainage fluid. Treatment with cefmetazole led to gradual shrinkage and eventual resolution of the abscess cavity. Following this, no recurrence occurred during 10 months of follow‐up.

## 3 Discussion

This case represents an extremely rare condition wherein cecal diverticulitis penetrated the retroperitoneum, leading to subcutaneous inguinal abscess formation. The mechanism of extension to the inguinal region is thought to be related to the anatomical structure of the retroperitoneum [1].

To the best of our knowledge, only one report has described a subcutaneous inguinal abscess from perforated cecal diverticulitis [3]. This difference may be attributed to variations in anatomical location and disease characteristics. Left‐sided colonic diverticulitis is more common in Western populations and tends to occur with a higher complication rate, whereas right‐sided colonic diverticulitis is more common in Asian populations and generally follows a milder course with fewer complications [4, 6]. To the best of our knowledge, this is the first report of laparoscopic surgery performed in a case of right‐sided colonic diverticulitis complicated by an inguinal abscess.

In a 10‐year observational study of right‐sided colonic diverticulitis, Hildebrand et al. reported that conservative treatment is feasible for uncomplicated cases without abscess or perforation [7]. However, in complicated cases involving such complications, surgical intervention is recommended. The study did not establish definitive criteria for the extent or timing of surgical resection, as these should be determined based on individual clinical presentations [7]. In this case, the initial treatment involved antibiotics and incision and drainage. After temporary improvement, recurrence prompted definitive surgery once inflammation had subsided. In this case, multiple diverticula were observed from the cecum to the ascending colon. A limited resection was considered; however, due to the extent of diverticulosis and inflammation, a right colectomy was selected to ensure complete removal and reduce recurrence risk. Although laparoscopic surgery was once contraindicated for diverticulitis, advances in the technique and accumulation of experience have made it safe and feasible even in complicated sigmoid cases [2]. Additionally, Schlussel et al. conducted a large‐scale analysis of surgical cases of right‐sided diverticulitis using the National Inpatient Sample database [8]. They reported relatively low rates of postoperative complications and in‐hospital mortality, with significantly fewer complications associated with laparoscopic surgery compared with open procedures. In their study, ileocecal resection and right colectomy were the most commonly performed procedures, supporting their efficacy as definitive treatment options for cases wherein conservative management is unsuccessful [8]. A stepwise strategy of conservative management followed by interval surgery, once the general condition is stabilized, is also applicable to the right colon and proved to be a realistic and effective option in this case.

The diagnosis of a fistula in this case required some ingenuity. Although a fistula was suspected on pre‐surgical contrast enema imaging, both the abscess and suspected tract diminished with conservative treatment, and intraoperative indigo carmine staining was unclear (Figure 4). However, the resected specimen showed staining of the cecal diverticulum, confirming the presence of a fistula and validating the resection.

Furthermore, a delayed postoperative complication was observed in the form of a residual abscess 5 months after the initial surgery. Although the primary lesion, including the fistulous cecal diverticulum, had been resected, chronic inflammation of the residual fistulous tract that extended from the retroperitoneum to the inguinal region was considered the cause. This highlights the importance of long‐term follow‐up in cases wherein extensive retroperitoneal and subcutaneous involvement is present. Conservative treatment with antibiotics and percutaneous drainage proved effective, suggesting that even delayed complications in such anatomically complex cases can often be managed without additional invasive procedures.

This case suggests that early imaging, local control of inflammation, and subsequent definitive laparoscopic surgery may be effective even in severe right‐sided colonic diverticulitis.

We report an exceptionally rare presentation of right‐sided colonic diverticulitis causing an inguinal subcutaneous abscess via retroperitoneal perforation, with extension to the skin, unprecedented globally. Considering the relatively high incidence of right‐sided diverticulitis in Asian countries, similar cases may be encountered in the future. Therefore, this stepwise treatment strategy may serve as a valuable reference in clinical practice.

This case report has several limitations. As a single case, it lacks generalizability and may not apply broadly to all patients with right‐sided colonic diverticulitis. The precise mechanism of abscess extension could not be conclusively established, and anatomical inferences remain speculative. Limited long‐term follow‐up data precluded the assessment of outcomes beyond the immediate postoperative period. These limitations suggest that further case accumulation and comparative studies are needed to validate the staged treatment strategy.

We encountered an extremely rare case of cecal diverticulitis that penetrated the retroperitoneum to form a subcutaneous inguinal abscess. A staged treatment approach, beginning with conservative therapy, followed by interval laparoscopic surgery, proved effective. This report provides useful insights into the treatment options and outcomes in patients with complicated right‐sided colonic diverticulitis.

## Author Contributions

All authors contributed to the diagnosis, management, and follow‐up of the patient. Koki Miya drafted the manuscript. All authors approved the final version of the manuscript and agree to be accountable for all aspects of the work.

## Ethics Statement

Ethics approval was not required for this case report in accordance with institutional policy.

## Consent

Written informed consent was obtained from the patient for publication of this case report and accompanying images.

## Conflicts of Interest

The authors declare no conflicts of interest.

## Data Availability

The data that support the findings of this study are available from the corresponding author upon reasonable request.
